# The efficacy of ginger for treating chemotherapy-induced nausea and vomiting in patients with cancer: a systematic review and meta-analysis

**DOI:** 10.2478/abm-2026-0030

**Published:** 2026-08-31

**Authors:** Min Li, Qianjun Li, Xuecheng Zeng, Misbah Ullah Khan, Wei Cai

**Affiliations:** Department of Pharmacy, Yibin Second Hospital of Traditional Chinese Medicine, Yibin 644000, China; Department of Outpatient, Yibin Second Hospital of Traditional Chinese Medicine, Yibin 644000, China; Center for Nanosciences, University of Okara, Okara 56130, Pakistan

**Keywords:** cancer, chemotherapy, cisplatin, ginger, nausea, vomiting

## Abstract

**Background:**

Ginger has been shown to effectively decrease the risk of nausea and vomiting in different medical conditions. In this meta-analysis, we investigated the effect of ginger on the risk of nausea and vomiting after chemotherapy.

**Methods:**

We searched the Cochrane Library, Scopus, PubMed, and Web of Science from inception to April 17, 2025, to identify randomized controlled trials assessing the effect of ginger on the incidence of nausea and vomiting after chemotherapy.

**Results:**

Finally, we included 13 randomized controlled trials with 639 participants in the ginger group and 626 participants in the comparator group assessing the efficacy of ginger for chemotherapy-induced nausea and vomiting. Overall, the risk of bias was generally low, with only 2 studies showing high risks of bias. We found that ginger could not significantly lower the risk of acute nausea (RR 0.83, 95% CI (0.60, 1.15), *I*^2^ = 38.35%) and acute vomiting (RR 0.73, 95% CI [0.51, 1.06], *I*^2^ = 0.00%) after chemotherapy. Similarly, ginger did not significantly decrease the risk of delayed nausea (RR 0.92, 95% CI [0.66, 1.28], *I*^2^ = 23.71%) and delayed vomiting (RR 1.17, 95% CI [0.75, 1.83], *I*^2^ = 17.43%) among those receiving emetogenic chemotherapy. In subgroup analysis, age was the only factor associated with the preventive effect of ginger on chemotherapy-induced nausea and vomiting, and ginger was more effective among those younger than 50 years.

**Conclusion:**

This meta-analysis indicated that ginger could not significantly alter the risk of nausea and vomiting after chemotherapy.

Nausea and vomiting are prevalent complications of chemotherapy with emetogenic agents, such as cisplatin, cyclophosphamide, and carboplatin, which can markedly undermine the quality of life and deter further treatment in severe cases [1, 2]. Data from 2,048 patients indicated that among those receiving single-agent highly emetogenic chemotherapy, 24% suffered from acute nausea, 49% suffered from delayed nausea, 8% experienced acute vomiting, and 14% experienced delayed vomiting [3]. In addition, among those receiving single-agent moderately emetogenic anti-cancer agents, 7% experienced acute nausea, 38% experienced delayed nausea, 2% experienced acute vomiting, and 14% experienced delayed vomiting [3]. A systematic review of 49 articles identified 28 patient-related risk factors that significantly affect the incidence of chemotherapy-induced nausea and vomiting [4]. Among these risk factors, history of nausea/vomiting, female sex, expectancy of chemotherapy-induced nausea and vomiting, younger age, anxiety, history of morning sickness, and low alcohol intake showed a stronger association with the risk of nausea and vomiting after chemotherapy [4].

Ginger and its active components have been shown to modulate several signaling pathways linked to nausea and vomiting, such as the substance P pathway and 5-hydroxytryptamine (5-HT)_3_ receptor pathway [5]. In addition, ginger was found to significantly lower the risk and severity of nausea and vomiting in pregnancy and among those undergoing surgery [6, 7]. Herein, several meta-analyses assessed the efficacy of ginger for nausea and vomiting among those undergoing chemotherapy; however, their results regarding the effect of ginger intake on the incidence of nausea and vomiting remain largely inconsistent, and a meta-analysis is needed to pool data from different studies [8,9,10,11,12,13]. Although there are some previous systematic reviews on the same topic, they included only 5–6 studies in their meta-analysis [14, 15], but this updated and comprehensive systematic review included 8–12 studies in the meta-analysis, providing more robust evidence for making recommendations.

There are significant differences and inconsistencies among the results of existing trials, making it challenging to achieve clear recommendations. Therefore, this meta-analysis was conducted to pool data from randomized controlled trials and investigate the effect of ginger on nausea and vomiting in the acute and chronic phases after chemotherapy.

## Methods

### Search strategy

We comprehensively searched PubMed, Scopus, the Cochrane Library, and Web of Science from their establishment to April 17, 2025, using the following search keywords: (“Ginger” OR “Zingiber officinale”) AND (“Chemotherapy”) AND (“nausea” OR “vomiting”).

The search strategy is presented in **Supplementary Material 1**. Furthermore, we checked the reference list of the identified trials or recent reviews to include more studies. The retrieved studies were transferred to EndNote 8.0, and duplicates were removed. This study was reported in compliance with the Preferred Reporting Items for Systematic Reviews and Meta-Analyses guideline [16], and the protocol of our meta-analysis was registered in PROSPERO (CRD42024613878).

### Inclusion and exclusion criteria

The inclusion criteria were as follows: (I) being a randomized controlled trial and (II) comparing the effect of ginger with placebo or absence of ginger on the incidence of acute (after 24 h) and delayed (after 72 h) nausea and vomiting after receiving emetogenic chemotherapy;

Exclusion criteria were as follows: (I) being an observational study, case report, or review article; (II) absence of a comparator group; (III) none of the target outcomes were reported; and (IV) ginger was used in combination with moxibustion.

### Data extraction and outcome measures

Two authors searched the literature and screened the eligibility of the identified studies. All discrepancies were resolved via discussion. By screening the titles and abstracts, the authors primarily assessed the relevance of the articles and assessed their compliance with the inclusion and exclusion criteria. Next, the full-text version of the remaining articles was assessed to check their eligibility for inclusion.

The following data were collected: study characteristics (the first author name, trial registry, study design and settings, publication year, and country of study), population (sample size, type of cancer, type of chemotherapy drug, sex, and age), interventions (dose of ginger and length of treatment), and the outcomes (the incidence of nausea and vomiting in the acute and chronic phases).

### Risk-of-bias assessment

Two researchers assessed the risk of bias for all studies independently, and all inconsistencies were resolved via discussions. We used the revised Cochrane risk-of-bias tool for randomized trials (RoB2) to investigate the risk of bias. The tool assesses the risk of bias based on the randomization process, missing outcome data, deviations from intended interventions, outcome measurement, and selection of the reported results and provides an overall risk of bias for studies. All domains received one of the following ranks: some concerns, low risk of bias, and high risk of bias [17].

### Statistical analysis

For all outcomes of interest, the number of those experiencing the outcome and the number of those not experiencing the outcome were extracted from the articles and used to calculate the pooled effect size. Subgroup analysis will be conducted based on patients and study characteristics, including sample size, age, gender, ginger dose, chemotherapy regimen, and concomitant use of antiemetic drugs, to identify the factors associated with the effect of ginger on chemotherapy-induced nausea and vomiting. Sensitivity analysis will be conducted using the leave-one-out test to assess the robustness of our findings. Publication bias will be assessed using funnel plot and Egger’s and Begg’s tests.

The chi-square test and *I*^2^ statistics were used to measure between-trial heterogeneity, and *I*^2^ more than 50% was regarded as high statistical heterogeneity. Due to significant methodological heterogeneity, a random-effects model (*DerSimonian*–*Laird*) was adopted to pool data. This meta-analysis was performed using Stata version 14.0 (StataCorp, TX, USA).

## Results

### The results of the systematic search

Initially, we identified 101 records in PubMed, 251 records in Scopus, 139 records in Web of Science, and 102 records in the Cochrane Library. Of the 593 articles, 132 were removed because of duplication. By screening the titles and abstracts, 419 articles were excluded due to the lack of relevance or noncompliance with the inclusion criteria. Next, the full text of 42 articles was meticulously assessed for their compliance with the inclusion and exclusion criteria. Finally, we included 13 randomized controlled trials with 639 participants in the ginger group and 626 participants in the comparator group (**Figure 1**). Except for 2 studies that compared the absence of ginger with ginger [18, 19], other studies compared a placebo group with a ginger group [8,9,10,11,12,13, 20,21,22,23,24]. Konmun et al. [22] administered the effective component of ginger, while others administered the ginger root powder. Ansari et al. [8], Li et al. [23], Manusirivithaya et al. [11], Shokri et al. [13], and Uthaipaisanwong et al. [24] included only women, but other studies included both men and women. Most studies applied ginger for 3–5 d, but some studies applied ginger during the chemotherapy cycle, extending its use for weeks [9, 22, 24]. The most common regimens of chemotherapy were cisplatin-based, doxorubicin-based, and platinum-based regimens. Furthermore, among the included studies, 2 studies [8, 22] did not use standard antiemetic agents and 2 studies [13, 18] did not report data on the use of standard antiemetic agents, while other studies reported that they concomitantly and equally administered antiemetic agents in both groups, which mostly included corticosteroids, 5-HT3 receptor antagonists, and neurokinin 1 (NK-1) receptor antagonists (**Table 1**).

**Figure 1.**
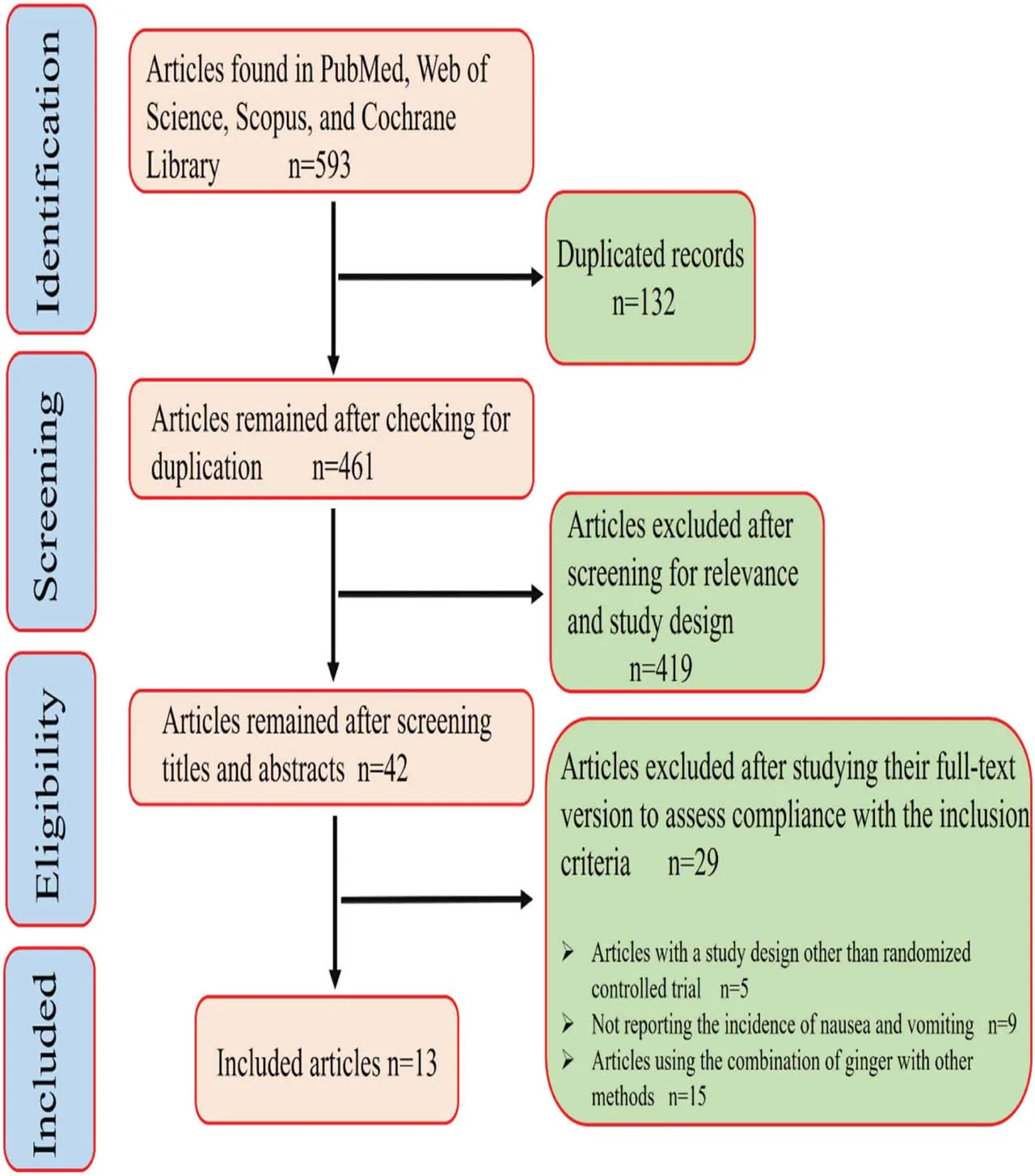
Systematic search flowchart.

**Table 1.** Study characteristics

**First author name**	**Publication year**	**Age (years, mean)**	**Country of study**	**Gender**	**Ginger dose**	**Length of treatment with ginger**	**Standard antiemetic regimen received in all groups**	**Number of participants in the ginger group**	**Number of participants in the comparator group**	**Type of cancer**	**Type of comparator**	**Type of chemotherapy**	**Reference**
Ansari et al.	2016	48.6	Iran	Women	500 mg twice per day	For 3 d	None	57	62	Breast cancer	Placebo	Doxorubicin-based chemotherapy	[8]
Bossi et al.	2017	59.1	Italy	Both	160 mg/d	The treatment ranged from 42 d to 56 d	Standard antiemetic prophylaxis for nausea/vomiting, including NK-1 receptor antagonists, 5-HT3 receptor antagonists, and dexamethasone	121	123	Mostly lung cancer and head and neck cancer	Placebo	High-dose cisplatin	[9]
Crichton et al.	2024	59	Australia	Both	1.2 g/d	Administered from the day of chemotherapy and continuing for 5 d for chemotherapy cycles 1 through 3	Standard antiemetic prophylaxis, including NK-1 receptor antagonists, 5-HT3 receptor antagonists, and dexamethasone	51	52	Heterogeneous	Placebo	Doxorubicin-based chemotherapy	[20]
Fahimi et al.	2010	50.53	Iran	Both	1 g/d	For 3 d	Standard antiemetic regimens, including 5-HT3 antagonist (granisetron) and corticosteroid (hydrocortisone)	36	36	Particularly lung cancer and solid tumors	Placebo	Cisplatin-based regimens	[21]
Konmun et al.	2017	53	Thailand	Both	6-gingerol 10 mg twice per day	Since 3 d before chemotherapy for 12 weeks	None	40	41	Solid tumors, mainly breast and ovarian cancer	Placebo	Heterogeneous, including anthracycline- and platinum-based chemotherapy	[22]
Li et al.	2018	57	China	Both	500 mg/d	For 5 d beginning on the first day of chemotherapy	Standard antiemetic therapy (5-HT3 receptor antagonists)	71	69	Lung cancer	Placebo	Cisplatin-based regimens	[23]
Manusirivithaya et al.	2004	46.5	Thailand	Women	1 g/d	For 5 d since the start of chemotherapy	Dexamethasone	43	43	Ovarian cancer and cervical cancer	Placebo	Cisplatin-based regimens	[11]
Panahi et al.	2012	51.8	Iran	Women	1.5 g/d	For 4 d from the initiation of chemotherapy	Granisetron plus dexamethasone	37	41	Breast cancer	Absence of ginger	Heterogeneous, such as docetaxel, epirubicin, and cyclophosphamide	[19]
Pillai et al.	2011	15.7	India	Both	1 g/d	From days 1 to 3 of the chemotherapy cycle	Ondansetron and dexamethasone	27	30	Bone sarcoma	Placebo	Cisplatin/doxorubicin-based chemotherapy	[12]
Shokri et al.	2017	52.7	Iran	Women	2 g/d	For 6 cycles of chemotherapy	Not reported	20	29	Ovarian cancer	Placebo	Platinum-based adjuvant chemotherapy	[13]
Zick et al.	2009	55.7	United States	Both	1 g/d and 2 g/d	For 3 d	5-HT3 receptor antagonists and/or aprepitant	83	46	Heterogeneous	Placebo	Heterogeneous	[10]
Uthaipaisanwong et al.	2020	53.9	Thailand	Women	2 g/d	Prescribed in odd cycles in the cross-over trial	Dexamethasone and ondansetron	23	24	Gynecologic cancer	Placebo	Carboplatin/paclitaxel	[24]
Kadhim et al.	2021	Not reported	Iraq	Both	1.5 g/d	For the first 5 d of the chemotherapy cycle	Not reported	30	30	Heterogeneous	Absence of ginger	Not reported	[18]

NK-1, neurokinin 1; 5-HT3, 5-hydroxytryptamine 3.

### Risk-of-bias assessment

The RoB2 indicated a high risk of bias for 2 studies [18, 19], which were conducted in an open-label manner. The risk of bias for other studies was generally low, and allocation concealment was the most common domain remaining unclear (**Supplementary Material 2**).

### Meta-analysis

Due to significant methodological heterogeneity among the included studies, we employed a random-effects model (DerSimonian–Laird) to conduct the meta-analysis. Pooling data from the included studies, we found that ginger could not significantly lower the risk of acute nausea (RR 0.83, 95% CI [0.60, 1.15], *I*^2^ = 38.35%) (**Figure 2**) and acute vomiting (RR 0.73, 95% CI [0.51, 1.06], *I*^2^ = 0.00%) (**Figure 3**). Similarly, ginger did not significantly decrease the risk of delayed nausea (RR 0.92, 95% CI [0.66, 1.28], *I*^2^ = 23.71%) (**Figure 4**) and delayed vomiting (RR 1.17, 95% CI [0.75, 1.83], *I*^2^ = 17.43%) among those receiving emetogenic chemotherapy (**Figure 5**).

**Figure 2.**
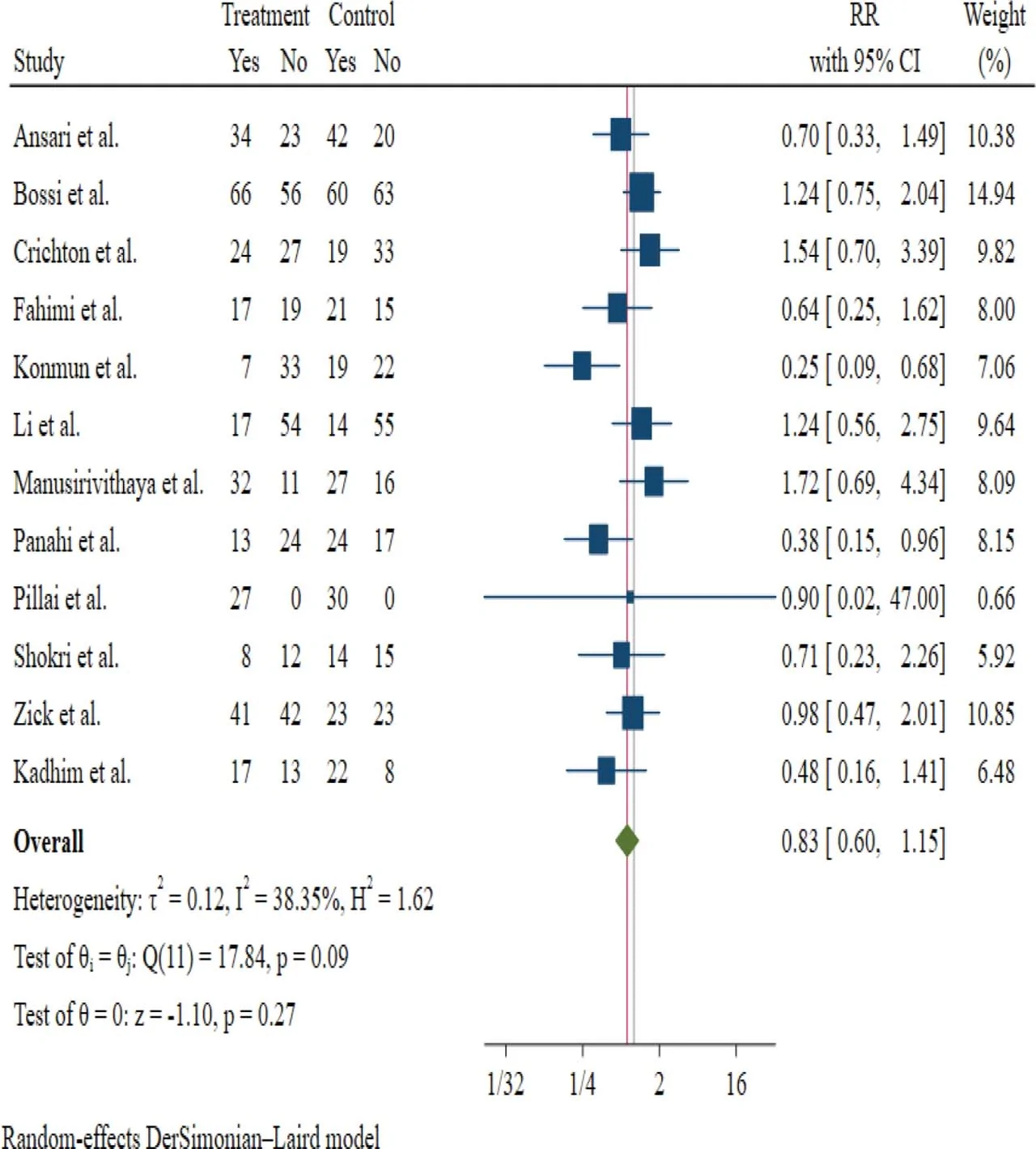
The effect of ginger on chemotherapy-induced nausea in the acute phase.

**Figure 3.**
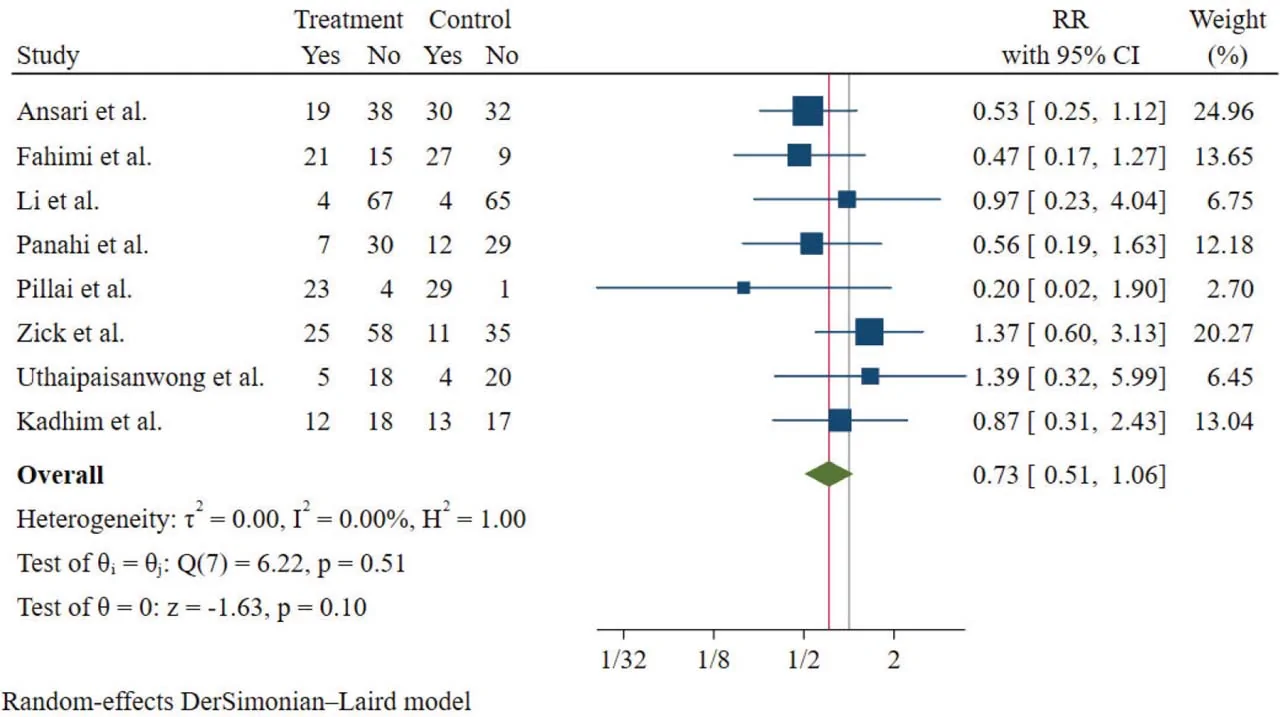
The effect of ginger on chemotherapy-induced vomiting in the acute phase.

**Figure 4.**
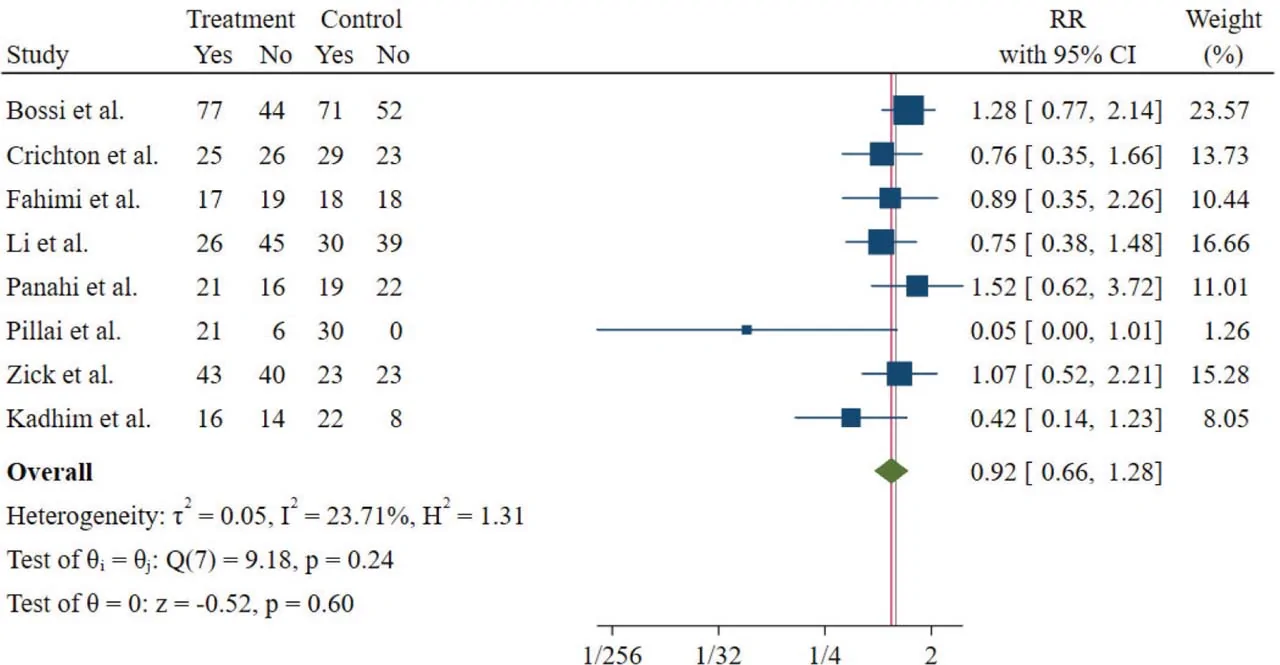
The effect of ginger on chemotherapy-induced nausea in the delayed phase.

**Figure 5.**
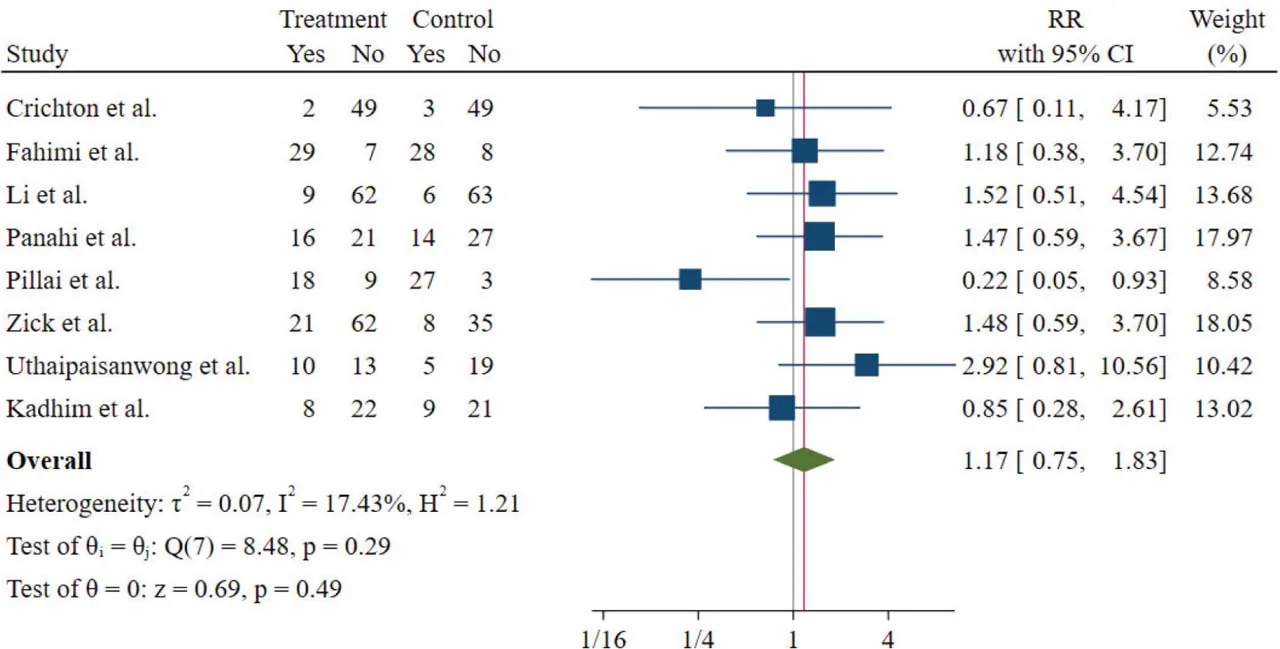
The effect of ginger on chemotherapy-induced vomiting in the delayed phase.

### Subgroup analysis

Subgroup analyses were conducted based on age, gender, dose of ginger, sample size, chemotherapy regimen, and concomitant use of standard antiemetic drugs. Among these variables, only sample size and concomitant use of standard antiemetic drugs significantly affect the results regarding acute nausea, showing that ginger was more effective in studies with a sample size less than 100 and in studies without concomitant use of standard antiemetic drugs compared to studies with a sample size greater than 100 and studies with concomitant use of standard antiemetic drugs. In addition, subgroup analysis indicated that the effect of ginger on acute nausea was nonsignificant in studies administering platinum-based regimens but significant in studies administering other regimens. Furthermore, only age could significantly affect the results regarding delayed nausea and delayed vomiting, showing that ginger was more effective in studies with participants’ mean age of less than 50 years compared to studies with a participants’ mean age of more than 50 years. However, none of the 4 variables could modulate the effect of ginger on vomiting in the acute phase (**Supplementary Material 3**).

### Sensitivity analysis

Using the leave-one-out test, we conducted a sensitivity analysis for nausea in the acute phase, the only variable that was reported in more than 10 studies. The results showed that the removal of any of the individual studies could not change the overall results, revealing the robustness of our findings (**Supplementary Material 4**).

### Publication bias

In visual inspection, the funnel plot showed degrees of asymmetry for all variables. However, Egger’s test indicated a significant risk of publication bias only for delayed nausea (Egger’s test *P* = 0.02, Begg’s test *P* = 0.10) but not for acute nausea (Egger’s test *P* = 0.26, Begg’s test *P* = 0.19), acute vomiting (Egger’s test *P* = 0.71, Begg’s test *P* = 0.90), or delayed vomiting (Egger’s test *P* = 0.17, Begg’s test *P* = 0.11) (**Supplementary Material 5**).

## Discussion

To our knowledge, this meta-analysis pooled data from the greatest number of studies to provide evidence for making more accurate recommendations. Pooling data from 13 studies, we found that ginger could not significantly decrease the incidence of nausea and vomiting in the acute and chronic phases after emetogenic chemotherapy.

Previous mechanistic studies have reported that ginger may lower the risk of nausea and vomiting by modulating the signaling mediated by 5-HT_3_, substance P, and acetylcholine receptor or by controlling gastrointestinal motility and gastric emptying rate [25]. In particular, the bioactive compounds of ginger, such as 8-shogaol, 8-gingerol, 6-shogaol, and 10-gingerol, are believed to mitigate acute vomiting by inhibiting the central and peripheral 5-HT system as the primary pathway inducing chemotherapy-induced nausea and vomiting [5]. Acute chemotherapy-induced nausea and vomiting, peaking at 5–6 h after chemotherapy, are mediated by 5-HT in the central nervous system and the gastrointestinal tract, but delayed chemotherapy-induced nausea and vomiting, peaking at 72 h after chemotherapy, are mediated by substance P in the central nervous system [26]. Besides, gingerols are believed to mitigate chemotherapy-induced nausea and vomiting partly by inhibiting dopamine synthesis and dopamine receptor 2 activation [27, 28]. Due to these similarities in the mechanism of action, clinical studies have increasingly investigated the effects of ginger on chemotherapy-induced nausea and vomiting. Although all the antiemetic properties of the active components of ginger and the emetogenic properties of chemotherapeutic agents seem to act through similar mechanisms, the antiemetic effects of ginger may not be strong enough to statistically produce a significant and meaningful impact in clinical practice. Similarly, not all antiemetic drugs can effectively prevent or control the emetogenic effects of chemotherapy. Furthermore, subgroup analysis showed that ginger more effectively reduced the incidence of acute nausea in studies not using standard antiemetic drugs compared to studies concomitantly administering standard antiemetic drugs. Since most of these drugs, such as 5-HT3 receptor antagonists, act through the same mechanisms through which ginger prevents nausea, additional inhibition of these pathways by ginger may not further enhance the antiemetic effect. This may explain the non-significant results observed in studies using standard antiemetic drugs.

Similar to our findings, another contemporary meta-analysis focusing on the effects of ginger on chemotherapy-induced nausea and vomiting indicated that ginger cannot significantly lower the risk of acute nausea, acute vomiting, delayed nausea, and delayed vomiting [14]; however, another meta-analysis with a smaller sample number of clinical trials of breast cancer showed that ginger can significantly lower the risk of acute nausea, acute vomiting, delayed nausea, and delayed vomiting [29]. Similar to our results, large-scale clinical trials, such as those conducted by Bossi et al. [9] and Li et al. [23], indicated that ginger is not superior to placebo in decreasing the risk of nausea and vomiting, and significant effects were observed only in some subgroups, such as among those with head and neck cancer [9].

In subgroup analysis, we found that ginger is more effective among those who are younger than 50 years. Consistently, it was previously shown that younger subjects are more susceptible to chemotherapy-induced nausea and vomiting [4]. Therefore, it can be hypothesized that ginger may be more effective in lowering the risk of chemotherapy-induced nausea and vomiting among those at a greater risk of such adverse effects, which is worth investigating in future randomized controlled trials. However, we observed no association between ginger dose or participants’ gender and the efficacy of ginger for preventing nausea and vomiting after chemotherapy.

Although this meta-analysis indicated that ginger cannot significantly reduce the incidence of nausea and vomiting after chemotherapy, the effect of ginger on the severity of chemotherapy-induced nausea and vomiting is another aspect that has been proven in previous meta-analyses [15]. Herein, a recent meta-analysis indicated that ginger could lower the risk of grade 3 chemotherapy-induced nausea and vomiting, but it could not significantly reduce the risk of low-grade chemotherapy-induced nausea and vomiting [15]. Therefore, it seems that ginger cannot decrease the risk of chemotherapy-induced nausea and vomiting but can effectively mitigate the severity of symptoms among those receiving emetogenic chemotherapeutic agents.

There are several limitations to our study. First, some of the studies had an open-label design, potentially undermining the accuracy of their results. Second, there were some methodological differences across studies. For example, the dose of ginger, the timing of treatment, and the length of treatment were not the same among studies, increasing methodological heterogeneity. Third, most clinical trials had a small sample size, reducing the reliability of their findings. Future large-scale clinical trials are expected to provide more data and assess our findings. Fourth, a considerable proportion of clinical trials on this topic did not report the incidence of nausea and vomiting and focused on other outcomes, which decreased the number of included studies for meta-analysis.

## Conclusion

This meta-analysis revealed that ginger did not significantly decrease the risk of acute or delayed nausea and vomiting after emetogenic chemotherapy. In subgroup analysis, age significantly affected the results regarding delayed nausea and delayed vomiting, showing that ginger was more effective in studies with a participants’ mean age of less than 50 years compared to studies with a participants’ mean age of more than 50 years. In addition, the effect of ginger on acute nausea was more effective in studies without concomitant use of standard antiemetic drugs. Future guidelines should weigh the pros and cons of treatment with ginger among this group of patients and make appropriate recommendations.

## Supplementary Material 1. Search strategy

### For PubMed

(“Ginger” OR “Zingiber officinale”) AND (“Chemotherapy”) AND (“nausea” OR “vomiting”)

### For Scopus

(“Ginger” OR “Zingiber officinale”) [title, abstract, keywords] AND (“Chemotherapy”) [title, abstract, keywords] AND (“nausea” OR “vomiting”) [all fields]

### For Web of Science

(“Ginger” OR “Zingiber officinale”) [title, abstract, keywords] AND (“Chemotherapy”) [title, abstract, keywords] AND (“nausea” OR “vomiting”) [all fields]

### For the Cochrane library

(“Ginger” OR “Zingiber officinale”) [title, abstract, keywords] AND (“Chemotherapy”) [title, abstract, keywords] AND (“nausea” OR “vomiting”) [all text]

## Notes

**Supplementary Material 2.** Risk of bias assessment based on the revised Cochrane risk-of-bias tool for randomized trials (RoB2).

**Trial/author name**	**Random sequence generation**	**Allocation concealment**	**Selective reporting**	**Blinding (participants and personnel)**	**Blinding (outcome assessment)**	**Incomplete outcome data**	**Other sources of bias**
Ansari et al.	Low	Unclear	Low	Unclear	Unclear	Low	Low
Bossi et al.	Low	Low	Low	Low	Low	Low	Low
Crichton et al.	Low	Low	Low	Low	Low	Low	Low
Fahimi et al.	Low	Unclear	Low	Low	Low	Low	Low
Konmun et al.	Low	Unclear	Low	Low	Low	Low	Low
Li et al.	Low	Low	Low	Low	Low	Low	Low
Manusirivithaya et al.	Low	Low	Low	Low	Low	Low	Low
Panahi et al.	Low	Low	Low	High	High	Low	Low
Pillai et al.	Low	Low	Low	Low	Low	Low	Low
Shokri et al.	Low	Unclear	Low	Unclear	Unclear	Low	Low
Zick et al.	Low	Low	Low	Low	Low	Low	Low
Uthaipaisanwong et al.	Low	Low	Low	Low	Low	Low	Low
Kadhim et al.	Low	Low	Low	High	High	Low	Low
